# Outbreak of Skin Lesions Among High School Wrestlers — Arizona, 2014

**Published:** 2015-05-29

**Authors:** Candice Williams, Jamie Wells, Ronald Klein, Tammy Sylvester, Rebecca Sunenshine

**Affiliations:** 1 Epidemic Intelligence Service, CDC; 2 Office for State, Tribal, Local and Territorial Support, CDC; 3 Disease Control Division, Maricopa County Department of Public Health, Arizona; 4 Career Epidemiology Field Officer, CDC

Skin infections are a common problem among athletes at all levels of competition; among wrestlers, 8.5% of all adverse events are caused by skin infections (1). Wrestlers are at risk because of the constant skin-to-skin contact required during practice and competition. The most common infections transmitted among high school wrestlers include fungal infections (e.g., ringworm), the viral infection herpes gladiatorum caused by herpes simplex virus–1 (HSV-1), and bacterial infections (e.g., impetigo) caused by *Staphylococcus* or *Streptococcus* species, including methicillin-resistant *Staphylococcal aureus* (MRSA) (2). On February 7, 2014, the Maricopa County Department of Public Health was notified of multiple wrestlers who reported skin lesions 2 weeks after participating in a wrestling tournament at school A. The tournament was held on January 24–25 and included 168 wrestlers representing 24 schools. The county health department initiated an investigation to identify cases of skin lesion, determine lesion etiology, identify risks associated with lesion development, and provide guidance for preventing additional cases.

Questionnaires were distributed to all wrestlers on teams that participated in the tournament and reported at least one skin lesion in a team member following the tournament. Medical records were obtained to verify lesion diagnosis where available. To include persons infected before and after the tournament, probable cases were defined as one or more skin lesions reported during January 1–March 1, 2014, by a wrestler who competed on a team that participated in the school A tournament. A confirmed case was a probable case with a physician-diagnosed skin lesion or laboratory-confirmation of a bacterial or viral infection of the skin.

A total of 47 cases (37 confirmed) were identified. Impetigo was the most common reported physician diagnosis (17 cases [46%]), followed by HSV-1 infection (11 [30%]), tinea corporis (two [5%]), and MRSA (two [5%]). One wrestler with physician-diagnosed HSV-1 reported having lesion onset 4 days before the January tournament and wrestling in the tournament with uncovered arm lesions. During the 2–9 days after the tournament, seven athletes who had wrestled in the tournament developed HSV-1 infection; during the 5–14 days after the tournament, three teammates who had not wrestled developed HSV-1. Another wrestler with physician-diagnosed impetigo reported having wrestled in the school A tournament with uncovered lesions on the head and neck. Subsequently, eight wrestlers who had participated in the tournament experienced impetigo 3–14 days after the tournament, and four teammates who did not participate in the tournament experienced impetigo 5–10 days after the tournament.

The Maricopa County Department of Public Health recommended that 1) wrestlers with visible, uncovered lesions be excluded from competition, 2) wrestling mats be disinfected between each match with a disinfectant approved by the Environmental Protection Agency as effective against MRSA and HSV-1, and 3) hand sanitizer be provided for use by all wrestlers during practices and competitions. In addition to implementing these recommendations, the Arizona Interscholastic Association also provided third-party clinicians who performed skin checks on each wrestler before competing.

This outbreak was caused by coincident spread of two distinct skin pathogens among high school wrestlers who had participated in the school A tournament. HSV-1 and impetigo caused by *Staphylococcus* or *Streptococcus* species were likely spread during the school A tournament by wrestlers who competed with uncovered lesions. CDC, the National Athletic Trainers’ Association, and the National Federation of State and High School Associations have each released statements and guidelines providing athletic staff and players with education regarding skin lesion prevention, lesion identification, and management (3,4). The *Journal of the American Osteopathic Association* also has published an evidence-based review with return-to-play guidelines for common dermatologic infections among athletes (5). This outbreak highlights the need for athletes, their coaches, and athletic directors to follow well-established infection control guidelines, including keeping all skin lesions covered with a clean, dry dressing, and excluding athletes from competitions when lesions cannot remain covered.
